# Network dynamics contribute to a gamma rhythm highly robust to synaptic variation

**DOI:** 10.1186/1471-2202-15-S1-P62

**Published:** 2014-07-21

**Authors:** Steven Hauser, Mark Reimers

**Affiliations:** 1 University of Virginia, Charlottesville, VA 22903, USA; 2 Virginia Institute of Psychiatric and Behavioral Genetics, Virginia Commonwealth University, Richmond, VA 23298, USA

## 

Dynamic homeostatic compensation in neural networks has recently attracted study. The gamma rhythm is often the focus of computational studies, as it is a local phenomenon and clinically significant: its disruption has been linked with mental illnesses such as schizophrenia and epilepsy. However little is known about dynamic compensation in relation to gamma rhythms. In (Reimers et al, in preparation) we observed that in all human brain genomics data sets, there is surprisingly high variability in mRNA levels for the key components of the GABA-A receptors, which are the mediators of fast inhibition. Further work showed that the variation was not compensated by substitution of different components of the same class of GABA-A receptor, suggesting at least a 20-fold variation in inhibitory signaling strength between individuals. Nevertheless most people seem to produce comparable gamma rhythms even with very different genetics.

We work with the Börgers-Kopell model, which has been used to demonstrate network mechanisms. They argue for a weak ‘PING’ model, in which the excitatory neurons play a role in maintaining oscillations as opposed to firing randomly between the regular spiking of inhibitory neurons. We find that their model is not very robust to large changes in synaptic parameter values (Figure 1A). We make a series of realistic modifications to the model, such as sparse connections, a lognormal distribution of connection weights, and a lowered inhibitory decay time constant [1]. We also lower the external drive in the model so that inhibitory neurons will not spike without excitatory input. Finally, we attempt to use more realistic synapses in our model, in which we fit model synapses to measured PSP data from physiological recordings.

**Figure 1:**
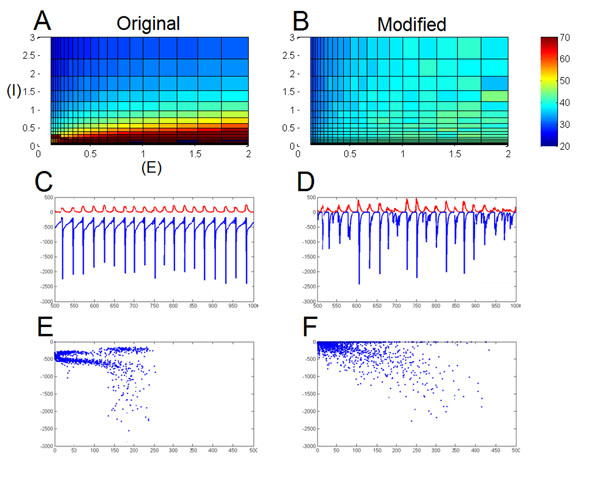
Figure 1 compares the original Börgers-Kopell ‘weak PING’ (left) to our modified version (right). **A.** and **B.** demonstrate the robustness of the models on gamma frequency over 100-fold inhibitory strength (Y) and 20-fold excitatory strength (X). **C.** and **D.** demonstrate the total *E*→*I* PSC (red) vs. the total *I*→*E* PSC (blue) during a simulation. **E.** and **F.** plot the correlation between the total PSCs.

## Conclusions

We find that the modified model is much more robust to variation in synaptic conductance, even on the scale suggested by transcriptomic variation (Figure 1A, B). Additionally, we find that each of our significant modifications increases the close tracking of inhibition to excitation during the gamma oscillation, in which we see a high correlation in our modified version (Figure 1D, F). This tracking seems to be the core of the robustness and has recently been shown to be an important modulator of the gamma rhythm [2]. The robustness of the modified model stems from network interaction rather than compensating parameters.
